# The Use of Iodoform

**Published:** 1885-05

**Authors:** 


### The Use of Iodoform.

"The reign of carbolic acid in Germany is over, and that of iodoform has begun," is the remark of a recent writer upon antiseptics, and in this sentence he expresses quite graphically the strong hold which this substance has obtained on the Continent. Scarcely less varied than the uses of cocaine are the cases in which iodoform has been recommended. We find it lauded as almost a specific in phthisis, and even hear of its successful administration in diabetes.

As a surgical dressing it seems to have superseded nearly every other—at least in certain clinics. One writer speaks highly of the treatment of old abscesses by injections of iodoform dissolved in glycerin, and another thinks compound fractures are best treated by sealing them up with the dry powder. Koenig is an enthusiastic advocate of the use of this substance in operations upon the joints, and introduces from seventy-five to a hundred and fifty grains into a cavity, apparently without inducing unpleasant general symptoms.

Iodoform suppositories in uterine surgery, and pencils of iodoform for fistulous tracts, have been deservedly recommended. Ligatures saturated with the drug have been employed with success.

Although iodoform has not been used in New York as widely and systematically as in Germany, its merits have been clearly recognized, especially in gynæcology, where its true sphere seems to lie. There are drawbacks with this as with almost every other antiseptic, but on the whole we may concede that the enthusiasm of foreign surgeons is not ill-founded.—*N. Y. Med. Journal.*

